# Phytochemical Synthesis of Silver Nanoparticles and Their Antimicrobial Investigation on Cotton and Wool Textiles

**DOI:** 10.3390/ma16113924

**Published:** 2023-05-24

**Authors:** Mihaela Cristina Lite, Roxana Constantinescu, Elena Cornelia Tănăsescu, Andrei Kuncser, Cosmin Romanițan, Dan Eduard Mihaiescu, Ioana Lacatusu, Nicoleta Badea

**Affiliations:** 1 Faculty of Chemical Engineering and Biotechnology, University Politehnica of Bucharest, 1-7, Polizu Street, 011061 Bucharest, Romania; cristina.lite@incdtp.ro (M.C.L.); cornelia.tanasescu@incdtp.ro (E.C.T.); dan.mihaiescu@upb.ro (D.E.M.); ioana.lacatusu@upb.ro (I.L.); 2 National Research and Development Institute for Textiles and Leather–INCDTP, Lucretiu Patrascanu 16, 030508 Bucharest, Romania; rodica.constantinescu@icpi.ro; 3 National Institute of Materials Physics, Atomistilor 405A, Magurele, 077125 Bucharest, Romania; andrei.kuncser@infim.ro; 4 National Institute for Research and Development in Microtechnologies, Erou Iancu Nicolae 126A, 077190 Voluntari, Romania; cosmin.romanitan@imt.ro

**Keywords:** antimicrobian activity, silver nanoparticles, *Stellaria media*, *Stellaria media*, textile fabrics

## Abstract

The use of bio-based reagents for silver nanoparticle (AgNP) production has gained much attention among researchers as it has paved the way for environmentally friendly approaches at low cost for synthesizing nanomaterials while maintaining their properties. In this study, *Stellaria media* aqueous extract was used for silver nanoparticle phyto-synthesis, and the resulting treatment was applied to textile fabrics to test its antimicrobial properties against bacteria and fungi strains. The chromatic effect was also established by determining the L*a*b* parameters. For optimizing the synthesis, different ratios of extract to silver precursor were tested using UV-Vis spectroscopy to observe the SPR-specific band. Moreover, the AgNP dispersions were tested for their antioxidant properties using chemiluminescence and TEAC methods, and the phenolic content was evaluated by the Folin-Ciocâlteu method. For the optimal ratio, values of average size, 50.11 ± 3.25 nm, zeta potential, −27.10 ± 2.16 mV, and polydispersity index, 0.209, were obtained via the DLS technique and zeta potential measurements. AgNPs were further characterized by EDX and XRD techniques to confirm their formation and by microscopic techniques to evaluate their morphology. TEM measurements revealed cvasi-spherical particles with sizes in the range of 10–30 nm, while SEM images confirmed their uniform distribution on the textile fiber surface.

## 1. Introduction

Since the promotion of the field of nanomaterials, many studies have reported their advanced properties in a vast range of applications. They have been used for medical applications [1], photocatalytic [2,3], sensors and biosensors [4,5], optoelectronics [6], etc. Silver nanoparticles (AgNPs) represent one of the most studied nanomaterials due to their versatility and remarkable antimicrobial properties. The antibacterial mechanism of AgNPs is related to their ability to interact with the sulfur contained in the proteins and amino acids of the bacterial cell membranes, leading to bacterial inactivation. Moreover, the released silver ion interacts with the phosphoric groups in the DNA, inhibiting the enzymes activities [7]. An important factor in antimicrobial effectiveness is the size of the nanoparticles. Panacek reported that nanoparticles of 25 nm diameter exhibit the most efficient antibacterial activity against bacteria compared to larger silver nanoparticles. [8].

One advantage of AgNPs is represented by the ease of producing them. The top-down approaches require the use of physical methods (such as laser ablation, ultrasonication, or ball milling), while the bottom-up methods involve the chemical or electrochemical reduction of silver ions [9]. Furthermore, with the advent of the green approaches, the drawbacks of the conventional methods (the high costs in the case of the physical methods and the hazardous reagents in the case of the chemical methods) were overcome, giving rise to a new field of research [10]. The literature on silver nanoparticle production reported the use of different green agents, such as bacteria, fungi, algae, yeasts, or plants [11]. Recent studies exploit the enhancement of silver nanoparticle bioperformance as a disinfection agent by designing AgNP biohybrids [12,13,14].

The plant-based synthesis of AgNPs originates from the reduction of silver ions by the phyto-constituents of the plants, such as metabolites and reductive biomolecules, including flavones, terpenoids, ketones, aldehydes, amides, polysaccharides, proteins, vitamins, etc. [15].

In the research of functional textiles, AgNPs have been studied in order to accomplish the antimicrobial property due to various factors such as stability, cost, and environmental-friendly considerations [7]. The syntheses were either performed in situ, by immersing the textiles in the reaction media while adding the reducing agent [16], or by coating the textile samples with the synthesized AgNPs [17]. Maghimaa and Veeraraghavan reported the production of AgNPs using *Curcuma longa* L. and *Scutellaria barbata*, respectively, with application in cotton fabric coatings for antimicrobial and wound healing activities [18,19]. The wound healing property originates from the inhibition of microorganisms causing infections [20]. Kabeerdass studied the antibacterial activity of silver nanoparticles synthesized by *Alternanthera sessilis* leaf extract and applied to textiles and determined zones of inhibition from 6 mm to 13 mm for *Escherichia coli*, *Staphylococcus aureus*, *Pseudomonas aeruginosa*, *Klebsiella pneumoniae*, *Klebsiella oxytoca*, and *Acinetobacter baumanii* bacterial strains [21]. Similar values (10 mm to 13 mm) were obtained by Eid et al. for *Streptomyces laurentii* mediated AgNP synthesis for developing antibacterial functional textile fabrics [22]. Another use of AgNPs for their antibacterial properties is in cultural heritage preservation for textiles [23], stone [24], paper [25,26], and parchment artifacts [27].

In this study, the green AgNP production mediated by *Stellaria media* aqueous extract is presented. *Stellaria media* is a medicinal plant widespread in Europe, Asia, North America, and Africa (cold and temperate regions), and it is known as chickweed. It is used for gastrointestinal disorders treatment, asthma, diarrhea, measles, and jaundice treatment, and for l, reproductive, and respiratory tract inflammations [28]. The phyto-constituents of *Stellaria media* are represented by flavonoids, such as apigenin derivatives (e.g., apigenin 6-C-arabinosyl-8-C-galactoside, apigenin 6-C-galactosyl-8-arabinoside, apigenin 6-C-glucosid, quercetin 3-O-rutynoside, etc.), and other elements: phenolic acids, pentasaccharides, lipids, triterpenic saponins (gypsogenine), fitosterols, coumarins, and vitamin C [29].

Different ratios of extract to silver ions were tested. The synthesis of the AgNPs was confirmed first by recording the UV-Vis spectra, and the efficiency of the reaction was demonstrated by the presence of the SPR band (surface plasmon resonance). For further optimization, the size (Z_ave_), polydispersity (PdI), and physical stability (evaluated by zeta potential measurements, ξ) were evaluated. Moreover, the prepared dispersions were tested for their antioxidant activity compared to the plant extract. Chemiluminescence and TEAC assays were performed in order to evaluate the ability of the dispersions to scavenge short- and long-lived free radicals, respectively (cationic radicals and oxygen species). The phenolic content was assessed for both the extract solutions and AgNP dispersions, and the polyphenol consumption was calculated. For the optimal ratio of extract to silver ions (*v*:*v*), the AgNPs were morphologically characterized by transmission electron microscopy (TEM), and their nature was confirmed by energy-dispersive X-ray spectroscopy (EDX). The crystallinity of the AgNPs was evaluated by X-ray diffraction (XRD).

A recent study reported the use of *Stellaria media* for producing silver and selenium nanoparticles [30]. Counting the previously mentioned aspects, the working hypothesis and novelty of the present research are represented by testing the *Stellaria media* mediated AgNPs for their antimicrobial and antioxidant profiles in several experiments, which include textile treatment. Thus, the cotton and wool samples were treated with AgNP dispersions, and antimicrobial effectiveness was tested against *Escherichia coli*, *Staphylococcus aureus*, *Bacillus subtilis* bacterial strains, and *Penicillium hirsutum* fungus strains. The morphological characteristics and the chromatic parameters of the treated textile samples were also evaluated and compared to those reported in the literature.

## 2. Materials and Methods

### 2.1. Materials

The reagents used in AgNP fabrication consisted of silver nitrate, purchased from Anal-R NORMAPUR, and *Stellaria media* dry plant from Anima Plant (a local producer). The reagents used in the analysis: Trolox (6-hydroxy-2,5,7,8-tetramethylchroman-2-carboxylic acid), potassium persulfate, ABTS (2,2azinobis-(3-ethylbenzthiazoline-6-sulfonic acid), anhydrous sodium carbonate, gallic acid, sodium chloride, hydrochloric acid, hydrogen peroxide, Tris (hydroxymethylaminomethane base), and luminol (5-amino-2,3-dihydro-phthalazine-1,4-dione)—were purchased from Merck (Darmstadt, Germany). The textile samples (cotton and wool) were provided by the National Research and Development Institute for Textiles and Leather (INCDTP). For the antimicrobial activity tests, *Escherichia coli* ATCC 10536, *Staphylococcus aureus* ATCC 6538, *Bacillus subtilis* ATCC 6633 bacterial strains, and *Penicillium hirsutum* ATCC 52,323 fungal strains were used, along with the following culture media: TSA (Casein Soya Bean), TSB (Tryptic Soy Broth), Digest Agar, NB (Nutrient broth), EA (Enumeration Agar), and SCDLP (Casein Soya Bean Digest).

### 2.2. Characterization of Stellaria Media Extract

The high-resolution mass spectrometry analysis was carried out using a Fourier transform-ion cyclotron resonance (FT-ICR) spectrometer, the SolariX XR 15T (Bruker Daltonics, Bremen, Germany). The sample was introduced by direct infusion. Positive ESI ionization was performed with a sample flow rate of 120 µL/h, a nebulization gas pressure (N_2_) of 2.2 bar at 180 °C, and a flow rate of 3.5 L/min. Negative ESI ionization was performed with a sample flow rate of 120 µL/h, a nebulization gas pressure (N_2_) of 2.8 bar at 180 °C, and a flow rate of 3 L/min. The spectra were recorded over a mass range between 46 and 1200 amu at a source voltage of 3900 V.

### 2.3. Synthesis, Optimization Strategies, and Physico-Chemical Characterization of Green Silver Nanoparticles

Silver nanoparticles were produced through a photo-catalyzed reaction, according to the synthesis procedure described in our previous work [31]. The reaction mixtures were incubated in a sunlight-simulating chamber for 24 h; afterward, they were transferred into brown containers due to the photosensitivity of the synthesis. Two concentrations of the plant extract were tested: 3 g/100 mL and 1 g/100 mL, respectively.

*Spectral optimization*. The optical properties of the silver nanoparticle suspensions prepared in different ratios of plant extract and silver precursor were evaluated by UV-Vis absorption spectroscopy using a Lambda 950 instrument from PerkinElmer (Waltham, MA, USA) on a spectral range of 200–700 nm. The electron density oscillation on the particle surface, induced by their nanometric dimensions, absorbs electromagnetic radiation. The presence of this oscillation, known as surface plasmon resonance (SPR), indicates the formation of AgNPs [32].

*Average size and polydispersity*. The resulting dispersions were characterized by dynamic light scattering (DLS) to determine the average size (Z_ave_) of the AgNPs (the hydrodynamic diameter) by measuring the intensity of the light scattered. A total of 300 µL of AgNPs dispersion were added to 20 mL of distilled water, and the resulting suspension was subjected to analysis. Using Zetasizer NanoZS (Malvern Instruments Inc., Worcestershire, UK) equipment, three sets of measurements were performed for each sample at 25 °C, and the mean values were reported. The polydispersity index was also determined from this analysis, representing the distribution of the particle populations as a function of size.

*Physical stability.* The physical stability of the AgNPs was reported in terms of zeta potential measurements. For sample preparation, 300 µL of AgNPs dispersion and 50 µL of NaCl 0.9% solution were added to 20 mL of distilled water. By applying an electric field across these suspensions using the Malvern Zetasizer Nano ZS (Malvern Instruments Inc., Worcestershire, UK), the zeta potential was determined. The samples were triple measured, and the mean values were reported.

*In vitro antioxidant activity.* The ability of the AgNPs dispersions to scavenge short- and long-life radicals was determined and compared to the extract solutions with respect to the preparation ratios of AgNO_3_. In order to make a correlation between the antioxidant activity and the phenolic content of the plant extract, the Folin-Ciocâlteu assay was performed for both plant extract solutions and AgNPs dispersions.

Chemiluminescence methods. To generate the short-lived oxygen free radicals (ROS), the reaction between luminol (0.01 mM) and H_2_O_2_ (0.01 mM) was performed using TRIS-HCl (pH 8.6) as a buffer solution. The intensity measured for this reaction (I_0_) represents the maximum CL intensity (blank sample), used as a reference [33]. From each sample, 100 µL were mixed with 200 µL luminol, 650 µL Tris-HCl buffer solution, and 50 µL H_2_O_2_. Chemiluminescence (CL) measurements were performed in triplicate using a Turner Design TD 20/20 USA chemiluminometer. For the tested samples, a maximum CL intensity (I_s_) was obtained, and the antioxidant activity (AA%) was calculated as follows:

(1) $$\%\mathrm{AA}=\frac{I_{0}-{\;I}_{s}}{I_{s}}\cdot 100$$

TEAC method. The long-lived radical 2,2′-azino-bis(3-ethylbenzothiazoline-6-sulfonic acid) diammonium salt radical cation (ABTS^●+^) was generated by performing the reaction between ABTS and potassium persulfate. The concentration of the reagents in aqueous solutions was 7 mM for ABTS and 2.45 mM for potassium persulfate. After 16 h, the reaction mixture was normalized by adjusting the absorbance value to 0.70 (±0.02) at 734 nm. Trolox standard solutions with a concentration between 0 and 60 µM were used to build the calibration curve (R^2^ = 0.9989). For the spectral measurements, a UV-Vis-NIR Spectrophotometer V670 instrument from Jasco (Tokyo, Japan) was used. The inhibition of the ABTS^●+^ radical was calculated as follows:

(2) $${\%\mathrm{Inh}\;\mathrm{ABTS}}^{\cdot +}=\frac{A_{0}-{\;A}_{s}}{A_{0}}\cdot 100$$

where A_0_ represents the absorbance of the reference solution (3 mL ABTS^●+^ normalized solution and 2 mL distilled water) and A_s_ is the absorbance of the tested samples (3 mL ABTS^●+^ normalized solution, 0.5 mL AgNPs dispersion/diluted extract, and 1.5 mL distilled water). All measurements were performed in triplicate, and the antioxidant activity was expressed as Trolox equivalent.

*Polyphenol content—FC method*. The phenolic content, expressed as gallic acid (GA) equivalent, was determined for the extract and the AgNPs dispersions by using the Folin-Ciocâlteu method, according to ISO 14502-1:2005 [34]. The extract solutions were prepared in the same ratios as the AgNP dispersions, however, by replacing the Ag+ precursor with distilled water. A total of 0.5 mL of each tested sample was mixed with a 4.5 mL solution of Na_2_CO_3_ anhydrous 7.5% (m/m) and 5 mL of Folin-Ciocâlteu reagent 10% (*v*/*v*). The mixtures were incubated for 1 h at room temperature in a dark place. The absorbance of each sample was recorded at λ = 765 nm in triplicate. Using gallic acid standard solutions with concentrations in the range from 0 to 60 µg/mL, a calibration curve was constructed (with R^2^ = 0.9997). The polyphenol’s average consumption was calculated by subtracting the polyphenol content in the AgNP dispersion from the phenolic content in the extract solutions.

*Morphological characterization of silver nanoparticles*. The optimal ratio (*v*:*v*) of extract to Ag^+^ precursor was selected for further experiments. The silver nanoparticles prepared with this ratio were characterized by performing high-resolution transmission electron microscopy using the Cs probe-corrected JEM ARM 200F analytical electron microscope (JEOL Ltd., Tokyo, Japan). The crystallinity of the AgNPs was evaluated by performing an XRD characterization (X-ray diffraction) using a 9 kW Rigaku SmartLab instrument (Rigaku, Tokyo, Japan), which was equipped with a Cu Kα_1_ source (λ = 0.154 nm). XRD patterns were recorded in a grazing incidence mode, with the incidence angle, ω fixed at 0.5, while 2θ varied from 35 to 80°.

### 2.4. Textiles Treatment and Characterization

The cotton and wool textile samples (10 × 10 cm) were immersed in the AgNPs dispersion prepared in the optimal ratio for 1 h at room temperature. Afterward, the samples were carefully squeezed to remove the excess liquid, and they were let to dry naturally overnight without undergoing any curing process.

*Morphologic characterization.* The treated and untreated textile samples were subjected to scanning electron microscopy (SEM) using an FEI Quanta 200 microscope (ThermoFisher Scientific, Waltham, MA, USA), which was equipped with an Everhart–Thornley (ET) detector. The measurements were performed at an accelerating voltage of 15 kV in low vacuum mode. The nature of the AgNPs deposited on the textile fibers was confirmed through X-ray dispersive spectroscopy (EDX) by using an X-ray detector from EDAX-AMETEK (Berwyn, PA, USA) along with the electronic microscope.

*Chromatic characterization*. The chromatic parameters, expressed using the CIE L*a*b* system of colors, were determined via a Datacolor instrument from DKSH Holding Ltd. (Zurich, Switzerland). Comparing the parameters for a control sample (untreated textile fabric) and for the treated samples, a total color change (ΔE*) can be calculated using the formula:

ΔE* = [(ΔL*)^2^ + (Δa*)^2^ + (Δb*)^2^]^1/2^ (3)

where the L* parameter represents the luminosity of the samples and has values in the range of 0–100 (0 for black and 100 for white). The a* and b* parameters refer to the color of the samples, and their values are situated between −100 and +100 [35].

*Antimicrobial characterization.* The antimicrobial effect of the AgNP dispersions was tested against bacterial (*Escherichia coli*, *Staphylococcus aureus*, and *Bacillus subtilis*) and fungal (*Penicillium hirsutum*) strains.

A qualitative assessment of the antibacterial activity was performed according to the ISO 20743: 2013 standard [36]. By using the absorption method, direct inoculation of the test bacteriological inoculum was performed on the treated samples. After 24 h of incubation, the colony-forming units (CFUs) on the plates were counted. The results were expressed as an average percentage. The bactericidal ratio, R (%), was calculated as follows:

(4) $$R\;\left(\%\right)=\frac{{\mathrm{CFU}}_{\mathrm{control}}-{\mathrm{CFU}}_{\mathrm{sample}}}{\begin{array}{c} {\mathrm{CFU}}_{\mathrm{control}} \end{array}}\times 100$$

where CFU_control_ represents the number of colony-forming units in the control sample and CFU_sample_ is the number of colony-forming units in the treated samples.

Additionally, to perform a qualitative assessment for both bacteria and fungi strains, the agar-well diffusion method was used [37,38]. A volume of each strain was spread on the entire Petri dish surface. The textile samples (10 mm in diameter) were placed in the center of the plates, on the surface of the nutrient medium. The plates were incubated at 37 °C for 24 h. The antimicrobial activity is indicated by the formation of a clear inhibition zone (IZ) around the sample. The inhibition zone was calculated according to the formula:

(5) $$\mathrm{IZ}=\frac{D-d}{2}$$

where D is the diameter of the textile sample plus the inhibition zone (mm), while d is the diameter of the textile sample (mm).

## 3. Results and Discussions

### 3.1. Characterization of the Plant Extract and Spectral Optimization of the AgNPs Dispersions

**Figure 1:**
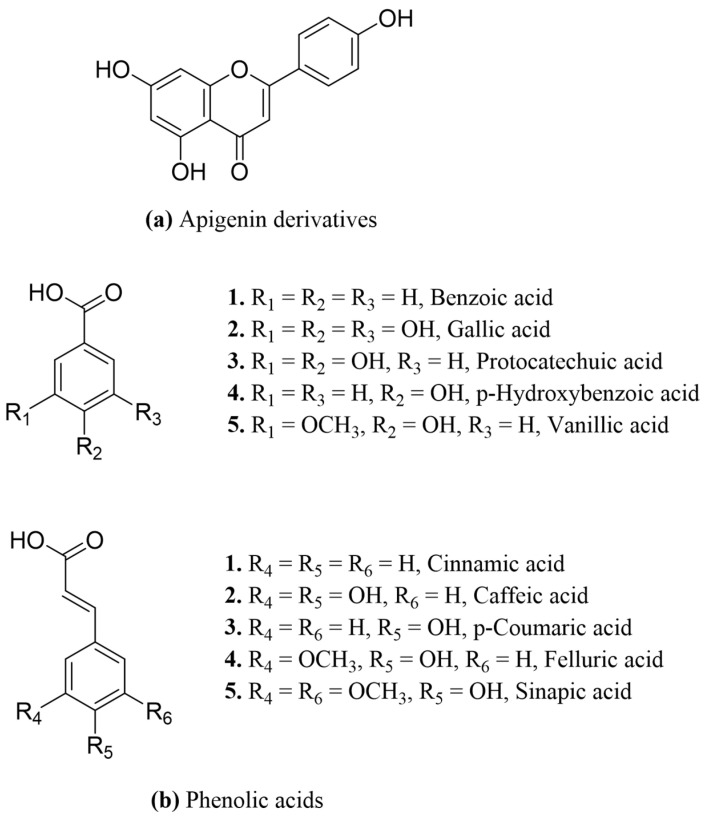
Major secondary metabolites from *Stellaria m.:* (**a**) flavonoids (with the basic skeleton of apigenin derivatives); (**b**) phenolic acids (non-flavonoid phenols).

The chromatographic characterization of phytochemical fractions of the *Stellaria media* plant has led to the identification of dozens of important bioactive metabolites. Most of the secondary metabolites identified to date belong to phenolic acids, flavonoids, triterpenoid saponins, and alkaloids [39]. Aside from these phytochemicals, the leaves of *Stellaria media* were reported to contain various proportions of carotenoids, g-linolenic acid, vitamins (e.g., A, B1, B2, B3, C, and E), amino acids, and essential dietary minerals (e.g., nickel, zinc, copper, sodium, cobalt, magnesium, iron, and manganese) [28]. Flavonoids and phenolic acids are the main types of phenolic compounds present in *Stellaria media* (Figure 1). Apigenin derivatives, which are flavonoids characterized by the presence of a ketone group between C-2 and C-3 and the attachment of the B ring to C-2, were widely distributed phytochemicals in *Stellaria media* [29].

**Figure 2:**
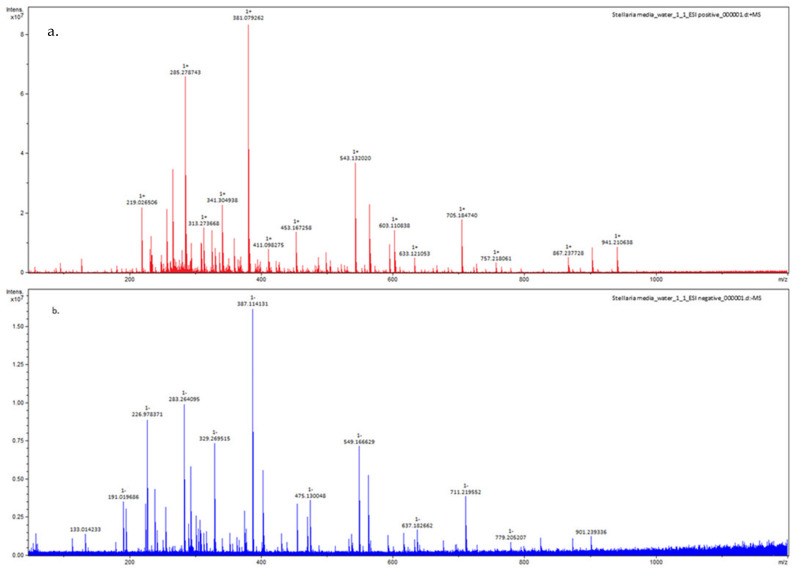
Entire mass spectra obtained of Stella Media on positive (**a**) and negative (**b**) ionization.

In the present study by high-resolution mass spectrometry (FT-ICR MS analysis), on both positive and negative ionizations (Table 1, Figure 2), several apigenin derivates were identified in aqueous *Stellaria media*. As presented in Table 1, the positive ionization allowed the identification of Apigenin 7-O-neohesperidoside (*m*/*z* 579.1706) and Apigenin-6-C-glucoside/Isovitexin (*m*/*z* 433.1129). These experimentally measured *m*/*z* data correspond to those of the theoretic *m*/*z* calculated mass (Table 1). Through both categories of ionization, positive and negative, apigenin-6,8-C-glucoside (595.1654 m/z/ESI+, 593.149 m/z/ESI-) and apigenin-6-arabynosyl-8-galactosyl (565.1549 m/z/ESI+, 563.1401 m/z/ESI-) were found in *Stellaria media*. The obtained monoisotopic MS spectra for ESI+ and ESI- can be found in the Supplementary Material (Figures S1–S4).

On the other hand, benzoic and cinnamic acid derivates in Figure 1b (e.g., vanillic, caffeic, gallic, p-hydroxybenzoic, chlorogenic, ferulic acid derivates, etc.) are part of the non-flavonoid phenolics and the main subgroup of phenolic acids distributed in *Stellaria media* [28]. Of these, four phenolic acids were identified by FT-ICR MS in the analyzed aqueous *Stellaria media*; their corresponding experimental cationic and anionic fragments were compared with the theoretically calculated ones (Table 1). For instance, the protonated molecular ions for ellagic and vanillic acid glucoside at experimental *m*/*z* 303.01, ESI+, and 353.08, ESI+, are in accordance with the theoretic *m*/*z* mass. Additionally, by negative fragmentation, the peaks at *m*/*z* 353.08/ESI—(caffeoylquinic acid) and hydroxybenzoic acid at *m*/*z* 137.024/ESI—were found to be consistent with those previously described by Gruz et al. in an analysis of phenolic acids from dietary polyphenols [40]. The MS spectra for ESI+ and ESI- of these phenolic acids can be found in the Supplementary Material (Figures S5–S8).

**Figure 3:**
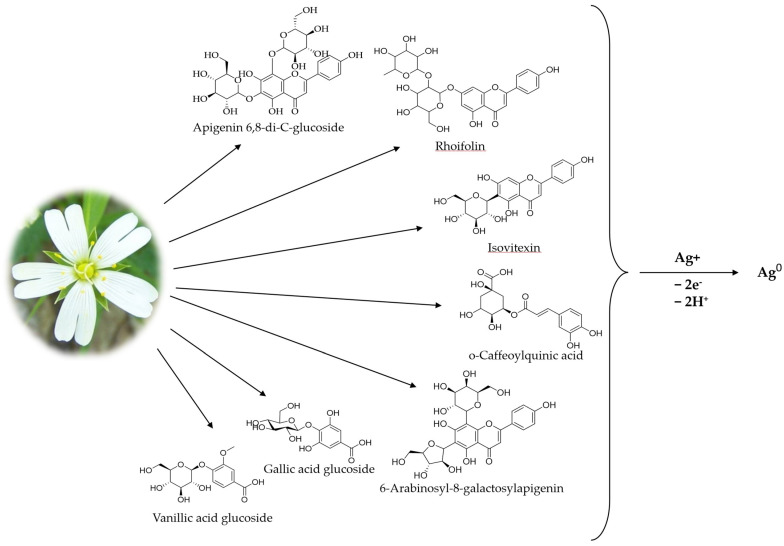
Schematic representation of AgNPs green synthesis using *Stellaria media* plant extract.

The phenolic compounds from both classes, flavonoids, and phenolic acids, identified in the plant extract played the role of reducing agents for the silver ions (Figure 3). Two concentrations of the plant extract were tested (1 and 3 g/100 mL). The volume ratio of the plant extract and the precursor was also varied, and after 24 h, the absorbance of the resulting dispersions was recorded. The first indicator of the successful reduction of the silver ions is the color of the dispersions, which become more brownish with time.

**Figure 4:**
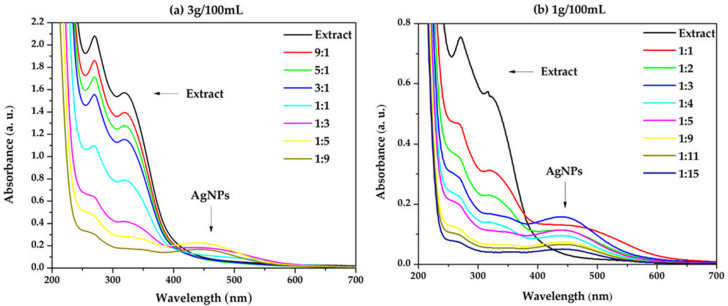
Overlapped UV-Vis absorption spectra recorded for the AgNPs dispersions at different ratios extract:AgNO_3_ (*v*/*v*) for extract concentrations (**a**) 3 g/100 mL and (**b**) 1 g/100 mL.

The presence of the SPR bands (surface plasmon resonance) in the UV-Vis spectra revealed the formation of the AgNPs. Figure 4a indicates that the formation of the nanoparticles is favored when increasing the silver precursor concentration in the reaction mixture. By further increasing the concentration of the silver precursor (Figure 4b), it is observed that the absorbance of the SPR band reaches a maximum at the 1:3 ratio (extract: AgNO_3_).

The aspect of the SPR band is strongly affected by the size and shape of the silver nanoparticles [41]. While some studies reported an increase in the SPR band intensity with the concentration of the plant extract, other authors found that it became broader and less intense. The increase in the SPR band intensity with the concentration of the plant extract is justified based on the increase in the reduction rate of Ag^+^ ions to Ag^0^, leading to a higher concentration of AgNPs [42]. On the other hand, the presence of large amounts of electron-rich phyto-molecules in the reaction medium causes the rapid reduction of Ag ions, leading to aggregation [43]. Considering these aspects along with the behavior of the SPR band in the present study, it is concluded that the phyto-composition of the plant extract used in the synthesis has a great influence on the shape of the SPR band. The subsequent experiments were carried out using an extract concentration of 1 g/100 mL.

### 3.2. Size and Polydispersity Optimization of the AgNPs Dispersions

**Figure 5:**
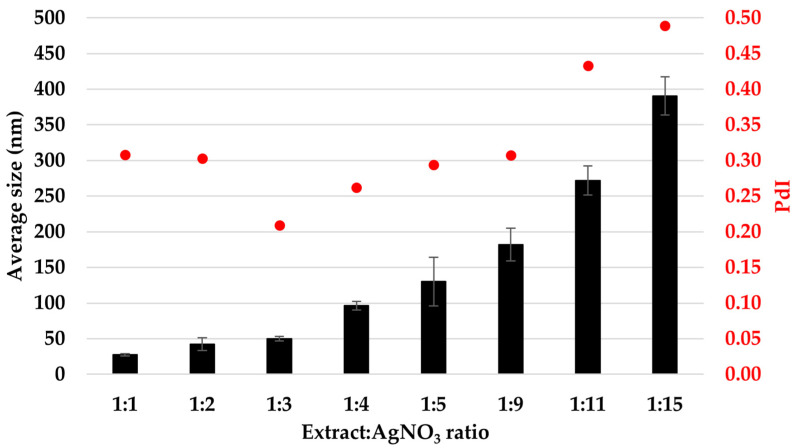
The average size (Z_ave_, black bars) and polydispersity index (PdI, red dots) of AgNPs, evaluated using the Dynamic Light Scattering (DLS) technique.

Figure 5 illustrates the hydrodynamic diameter expressed as the average particle size (Z_ave_) and the polydispersity indices (PdI) of the silver particles. A general trend of increasing the average particle size with increasing silver precursor concentration is observed, from 27.42 ± 1.64 nm for 1:1 to 390.60 ± 26.81 for 1:15 ratios. On the other hand, the polydisperse index varies in the range of 0.2–0.3 for the ratios 1:1 to 1:9, reaching a minimum of 0.209 for the ratio 1:3, and it is increased to 0.4–0.5 for the ratios 1:11 and 1:15.

When correlating the average size values of the AgNPs obtained by the DLS technique with the UV-Vis overlapped spectra, the trend of increased particle sizes follows the decrease of the SPR band. The exception to this trend is the dispersion prepared with the ratio 1:3. This exception is attributed to the low value of the PdI index recorded for this ratio, which indicates that a better homogeneity of the dispersion determines a more intense and sharper SPR band in the UV-Vis spectrum. The average particle size obtained for the ratio 1:3 is 50.11 ± 3.25 nm. Similar values were obtained when *Mentha piperita*, *Polyalthia longifolia*, *Swietenia mahogany*, or *Moringa oleifera* were used for producing AgNPs [44,45].

### 3.3. Physical Stability of the AgNPs Dispersions

**Figure 6:**
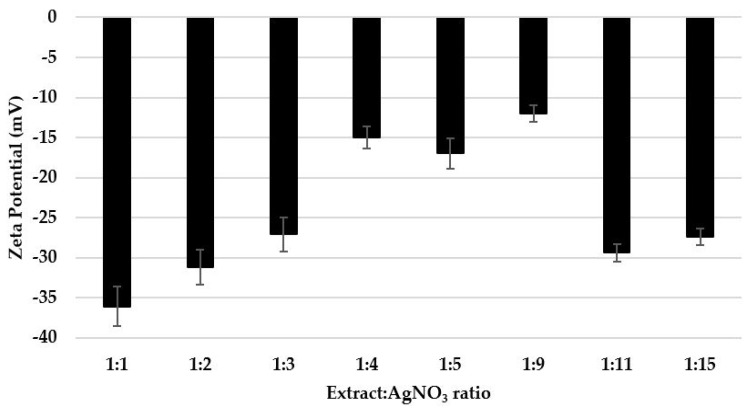
Zeta potential measurements of the AgNPs dispersions.

The stability of silver nanoparticles was evaluated by measuring the zeta potential (ξ). Based on these values (Figure 6), it was found that the dispersions prepared with the ratios 1:1, 1:2, 1:3, 1:11, and 1:15 present moderate stability, with zeta potential values in the range from −27.1 to −36.1 mV. On the other hand, the dispersions with the ratios 1:4, 1:5, and 1:9 are physically unstable (ξ is in the range from −12 to −17 mV), and they tend to segregate in time.

The values of the zeta potential do not follow a clear pattern when the precursor ratio is increased, proving that the stability of the dispersions is determined by a cumulative number of factors, such as the average size, the polydispersity, the nature of the phytoconstituents of the dispersion medium and their concentration, etc. [46]. The values of the zeta potential are similar to those reported for AgNPs synthesized using *Mentha piperita*, *Amaranthus retroflexus*, and pectin extracted from lemon peels [47].

### 3.4. In Vitro Antioxidant Activity and Phenolic Content Assessment

The tested plant extract solutions were prepared using the same ratios as the AgNP dispersions by replacing the precursor with distilled water.

#### 3.4.1. Chemiluminescence Assay

**Figure 7:**
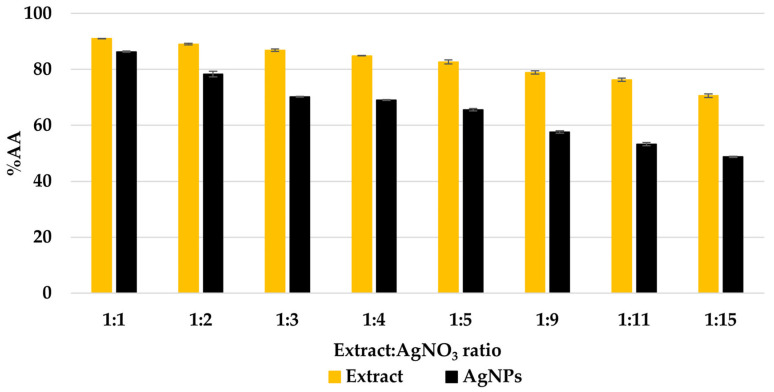
Chemiluminescence profile of the extract solutions and the AgNPs dispersions.

The undiluted extract solutions and AgNPs dispersions exhibited a strong capacity to scavenge short-lived free radicals (%AA > 99%). In order to achieve a quantifiable difference between the extract solutions and AgNP dispersions, all samples were diluted 10 times. The profile of the CL variation (Figure 7) shows a decrease in the antioxidant activity of the AgNP dispersions compared to the extract solutions. However, the diluted AgNP dispersions provided inhibition percentages in the range of 50–86%.

The variation of the chemiluminescence correlates with the results obtained in our previous study [31]. This behavior is attributed to the AgNPs’ ability to scavenge short-lived free radicals, which is higher compared to that of the AgNPs.

#### 3.4.2. TEAC Method

**Figure 8:**
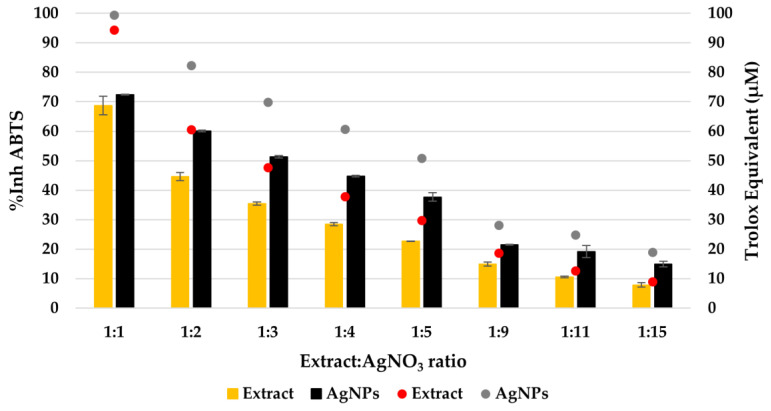
The antioxidant activity (AA%) of the extract solutions (red dots) and AgNPs dispersions (gray dots). The inhibition ABTS radical of the extract solutions (yellow bars) and AgNPs dispersions (black bars) evaluated by TEAC method.

By evaluating the ability of the extract solutions and AgNPs dispersions to scavenge long-lived cationic radicals, a different behavior was observed compared to the CL assay. The TEAC method revealed that the antioxidant activity was increased for the AgNP dispersions compared to the extract solution (Figure 8). The calculated Trolox equivalent of the pure extract was 17.6 ±1.7 µmol/g of the dry plant.

The Trolox equivalent varies not only from plant to plant but also from one plant part to another [48]. When synthesizing AgNPs with *Stellaria media* extract, the capacity to scavenge the ABTS long-lived free radicals is increased due to the presence of AgNPs. The same behavior was determined for silver nanoparticles obtained by using diverse varieties of *Cannabis sativa* leaf extracts [49]. On the other hand, when using *T. vulgaris* extract, a decrease in the Trolox equivalent was observed [50], suggesting that the antioxidant properties are influenced by the phyto-synthesized AgNPs. The enhancement of antioxidant activity in the presence of AgNPs is attributed to a synergistic effect between the plant constituents and the AgNPs, causing the nanoparticles to act as hydrogen donors and singlet oxygen quenchers [51].

#### 3.4.3. Phenolic Content Assessment

**Figure 9:**
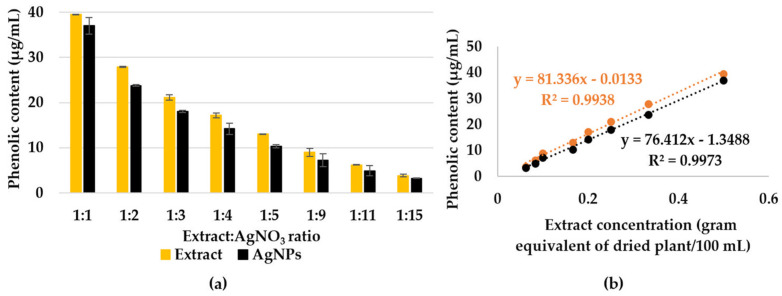
(**a**) Phenolic content of the extract solutions and AgNP dispersions, evaluated by Folin-Ciocâlteu method. (**b**) The phenolic content dependency on the extract concentration in the extract solutions (orange) and AgNPs dispersions (black).

By determining the phenolic content in the extract solutions and AgNPs dispersions (Figure 9a), a relationship of direct proportionality was observed between the extract concentration and total polyphenols (Figure 9b). Based on this relationship, it was concluded that the same amount of polyphenols is consumed in the reaction for all ratios. Hence, phenolic consumption was calculated. The phenolic content in the extract, calculated and expressed as GA equivalent, is 7.95 ± 0.84 mg/g of the dry plant. The calculated consumption was 12.83 µg/mL (representing 16.1% of the total amount of polyphenols).

The amount of phenolic content is comparable to that obtained for *Oudneya Africana*, *Thapsia garganica*, and *Terminalia arjuna* [52,53]. Moreover, similar consumption percentages were obtained for silver for nanoparticles synthesized with *A. absinthium* (17.5%) and *T. vulgaris* (21.2%) [50].

### 3.5. Silver Nanoparticles Characterization

The optimal ratio of extract to silver ions was 1:3 (extract concentration 1 g/100 mL), as it provided good physical stability (ξ = −27.10 ± 2.16 mV) when silver nanoparticles with an average size of 50.11 ± 3.25 nm were obtained. Moreover, the polydisperse index reached its minimum value (0.209) when this ratio was used. The AgNPs produced using this ratio were subjected to further characterization by TEM, EDX, and XRD techniques.

**Figure 10:**
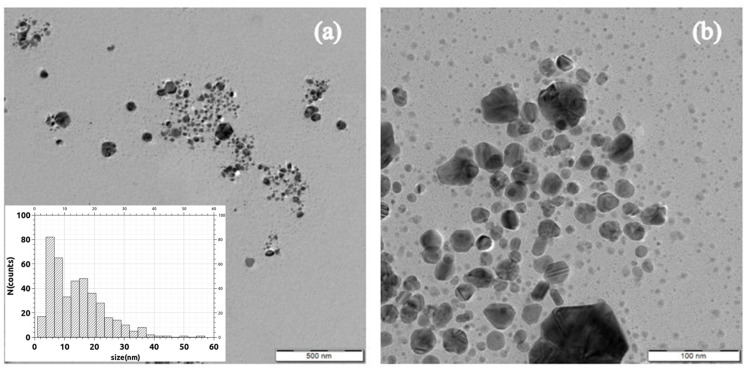
TEM images of AgNPs synthesized using *Stellaria media* extract 1:3 (**a**) 500 nm scale and (**b**) 100 nm scale. Inset of (**a**) shows the size distribution of dominant morphologies (cvasi-spherical, <100 nm).

The AgNPs present several types of morphologies (Figure 10), showing a system of defected AgNPs (roughly 100 nm) and two additional systems of finer, cvasi-spherical AgNPs (30 nm range and smaller than 10 nm, respectively). Larger formations (>1 µm) that show crystal texture randomly appear, but the systems of finer, cvasi-spherical silver NPs appear to be dominant.

As expected, the particle diameter measured by TEM is smaller than that determined by DLS for the dispersion in the ratio 1:3. This difference was observed in other studies regarding nanoparticle synthesis [54], and it is attributed to the fact that the DLS technique measures the hydrodynamic diameter of the particles [55].

**Figure 11:**
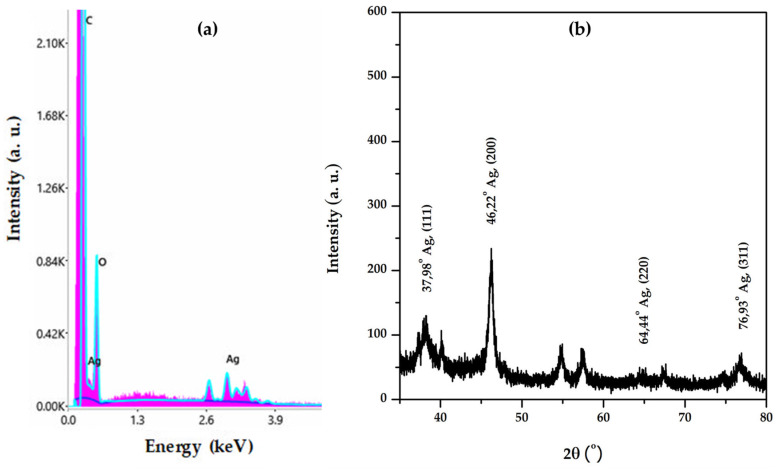
(**a**) EDX spectrum of AgNPs synthesized using *Stellaria media* extract and (**b**) XRD diffractogram of AgNPs synthesized using *Stellaria media* extract.

The nature of the particles was confirmed by energy dispersive X-ray spectroscopy. In the EDX spectrum (Figure 11a), the peak corresponding to Ag is typically present at 3 keV [56]. The other elements present in the EDX spectrum originate from the phytogenic components of the plant extract.

The pattern of the diffractogram obtained (Figure 11b) corresponds to the diffraction lines of different planes of silver nanoparticles: (111) at 2θ = 37.98°, (200) at 2θ = 46.22°, (220) at 2θ = 64.44°, and (311) at 2θ = 76.93°, with a crystalline packing of cubic silver (a = 0.41 nm, 225: Fm3m spatial group). Additional diffraction lines were also observed in the diffractogram, which correspond to NaCl and could originate from the washing water. The results are analogous to those obtained when using *Myrsine africana*, *Urtica dioica* leaves, and *Vitis vinifera* fruit extracts [57,58].

### 3.6. Characterization of the Treated Textile Samples

The cotton and wool samples treated with the AgNPs dispersion prepared in the optimal ratio (1:3) were characterized in terms of morphological, chromatic, and antimicrobial activity.

#### 3.6.1. Morphological Characterization of the Treated Textile Samples

**Figure 12:**
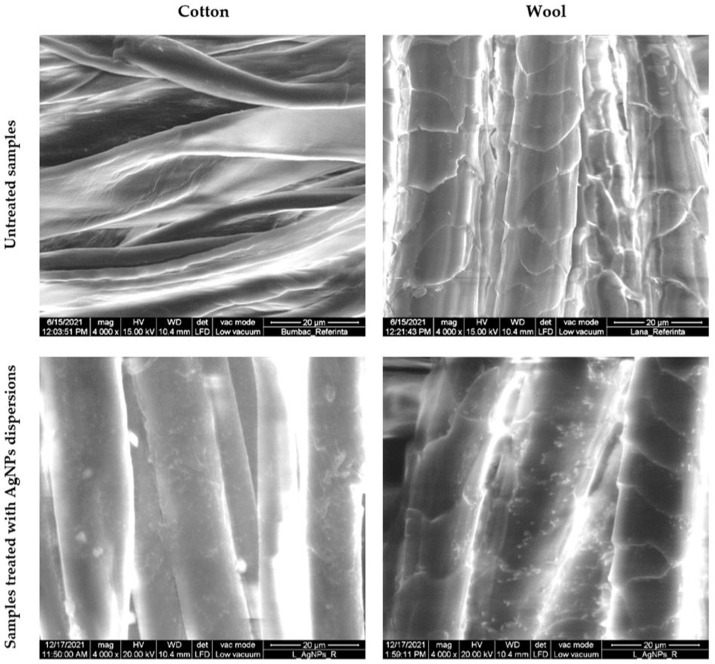
SEM images of textile samples treated with AgNPs dispersions synthesized using *Stellaria media* extract.

**Figure 13:**
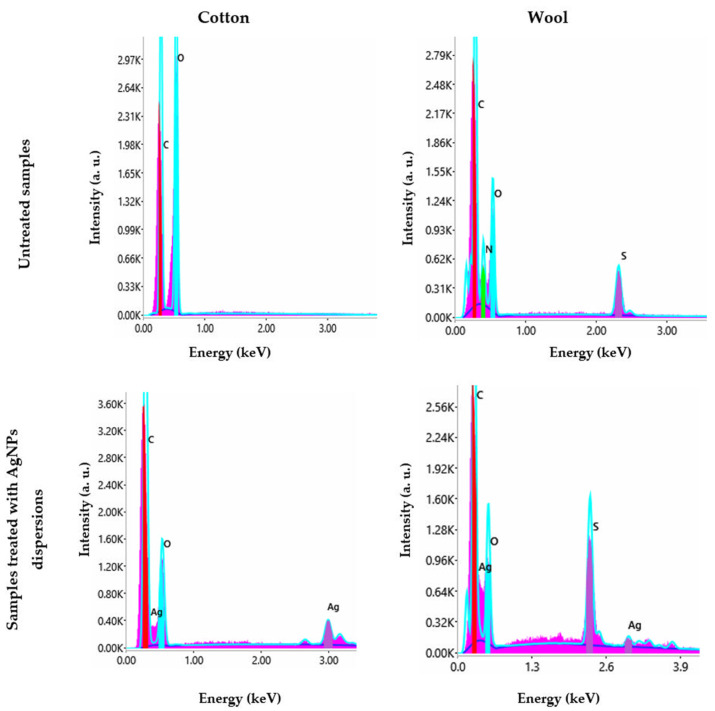
EDX spectra of textile samples treated with AgNPs dispersions synthesized using *Stellaria media* extract.

The distribution of the produced AgNPs on the textile fibers was evaluated by SEM characterization (Figure 12). EDX was performed to confirm their nature (Figure 13). The results were compared to the samples. The micrographs revealed that the AgNPs adhered uniformly to the surface of the textile fiber without deteriorating it.

SEM images show a uniform covering of nanoparticles that adhered to the surface of the fibers without producing any damage to their integrity. EDX spectra revealed the signal of silver for both cotton and wool samples. The results are correlated with other publications [59,60].

#### 3.6.2. Chromatic Parameters of the Treated Textile Samples

**Figure 14:**
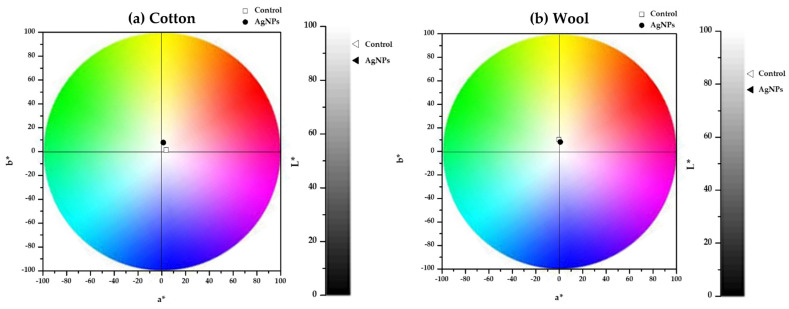
Chromatic diagrams of the textile samples treated with AgNPs dispersions (**a**) cotton and (**b**) wool.

With the measured L* a* b* parameters listed in Table 2, the total color shift was calculated from the control to the treated one. The values of ΔE* were similar for the two types of textile samples (7.33 for cotton and 6.50 for wool). Figure 14 illustrates the color change of the textile samples and indicates a slight shift of the color towards yellow for the cotton sample, while for wool, the color is insignificantly changed.

Compared to our previous study, the value of the total color change obtained for cotton was lower when *Stellaria media* extract was used for the synthesis, while the wool sample presented a similar value [31]. In addition, when compared to other studies, the total color change obtained in the present work is considerably lower. When treating cotton with AgNPs produced using the biomass filtrate of the *A. alternata* fungus strain, the color shift values varied from 13.4 to 41.8 [61]. In the case of wool samples, Sakil Mahmud determined a color change of 7.47 when treating the textile with 35 ppm AgNPs [62]. The most affected parameters are the luminosity and b* parameters, meaning that in most cases, the samples acquire a yellow hue and tend to become darker [63,64].

#### 3.6.3. Antimicrobial Activity Assessment

By counting the colony-forming units (CFU) of the bacteria strains, bactericidal ratios exceeding 99.99% were obtained for both types of textiles (Table 3). These percentages demonstrate that there was no contamination with *E. coli* for either of the textile samples, while the contamination with *S. aureus* was minimal. In the case of *B. subtilis*, only 2 CFU were tracked on the wool sample, while the cotton sample presented a 100% bactericidal ratio.

**Figure 15:**
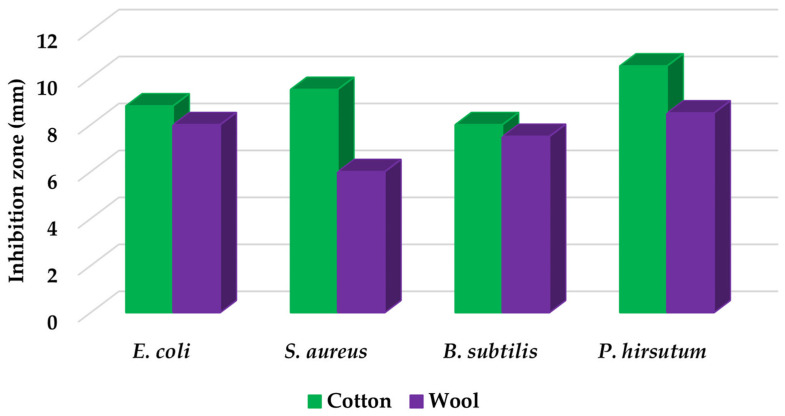
Inhibition zone of the microbial strains inoculated on the plates containing textile samples treated with AgNP dispersions.

Table 4 contains the qualitative assessment pictures of clear zones of inhibition. The dimensions of the IZ are compared in Figure 15.

The bacterial reduction exceeded 99.99% in all cases. These results are superior to those reported by Katarzyna Pietrzak for cotton fabric treated with silver nanoparticles (bacteria reduction up to 99.95%) [65] and by Abdelghaffar F. and colab. for linen fabrics treated with green synthesized silver nanoparticles (up to 98.45%, which was maintained up to 91.20% after 20 cycles of washing) [66]. For all microbial strains, higher values of inhibition zones were determined on the cotton samples compared to the wool samples. This observation is complementary to our previous publication, where better values were obtained for wool samples in the case of bacteria strains. The first conclusion is that the AgNPs dispersion fabricated with *Stellaria media* constitutes a more suitable antimicrobial treatment for cotton samples. Comparing the inhibition zone values, a slightly higher effectiveness was observed against the fungal strain (8.5 mm for wool and 10.5 mm for cotton) compared to the bacterial strains. Although the difference is not a significant one between the values of the inhibition zones in the case of the two categories of bacteria and fungi (e.g., 9–11 mm for bacteria vs. 12 mm for fungi in the cotton sample), the slightly higher effectiveness against fungi than bacteria could be associated with the cell difference between them. Bacteria, being prokaryotic organisms, have less complexity in cell structure, while fungi, as eukaryotes, have much more complex cellular organization. The fungal cell membrane could effectively withstand the action of AgNPs. A potential better penetration of AgNP in the bacteria’s membrane lipid bilayers than in those of fungal cells could be responsible for the cell lysis. Another assumption could be related to the fact that AgNPs better surround the bacterial pathogen and inhibit its pivotal functioning by entering the cell. This hypothesis is in line with a study developed by Mansoor et al., who ascribed that AgNPs cause protein denaturation, promote lipid peroxidation, and damage the fungal cell wall [67]. Additionally, Kim KJ et al. reported that silver nanoparticles dissipating the electrical potential of the membrane break down the bacterial membrane permeability barrier and cause cellular leakage [68].

The antibacterial activity determined on the cotton textiles varied in the following order: *Saphylococcus aureus* > *Escherichia coli* > *Bacillus subtilis*, and for wool samples: *Escherichia coli* > *Saphylococcus aureus* > *Bacillus subtilis*. All values were maintained in a narrow range (from 6 mm to 10.5 mm) and are higher than those obtained by Abdelghaffar F. (2021), which oscillates around 3.7 mm, or by Salem S.S. (2020), with values from 1.3 mm to 2.7 mm [66,69].

## 4. Conclusions

Ag-nanoparticles have been produced by a green Stellaria media mediated phytosynthesis. Both flavonoids and phenolic acids from plant extracts played a significant role in reducing the silver ions. The phenolic consumption for the reduction reaction was calculated and expressed as gallic acid equivalent, i.e., 12.83 µg/mL (representing 16.1% of the total amount of polyphenols). The phyto-composition of the plant extract used in the synthesis has a great influence on the shape of the SPR band. The formation of the AgNPs was favored by increasing the silver precursor concentration in the reaction mixture, i.e., the absorbance of the SPR band reaches a maximum at a ratio between extract and AgNO3 of 1:3. Optimized AgNPs had an average size of 50.11 ± 3.25 nm a zeta potential of −27.10 ± 2.16 mV and a cvasi-spherical morphology. The chemiluminescence antioxidant profile has underlined that the diluted AgNP dispersions provided inhibition percentages of oxygen radicals that ranged between 50% and 86%. This AgNP manifested an effective capacity to scavenge the ABTS long-lived free radicals, attributed to a synergistic effect between the plant constituents and the AgNPs. The cotton and wool textiles treated with the AgNPs were characterized in terms of morphological, chromatic, and antimicrobial activity. The micrographs revealed that the AgNPs uniformly adhered to the textile fiber surface without producing any damage to their integrity. The chromatic effect was highlighted by determining the chromatic parameters of the treated textile samples, expressed in the CIE L*a*b* system. The results obtained revealed that both textiles have undergone minor color changes, i.e., a slight shift of the color towards yellow, for the cotton sample, while for wool, the color was insignificantly changed. Stellaria media—Ag-NPs applied to textile fabrics presented superior performances in terms of antimicrobial activity against bacteria and fungi strains. For all microbial strains, higher values of inhibition zones were determined on the cotton samples compared to the wool samples. Thus, it can be concluded that green Stellaria media-mediated AgNPs could represent an attractive antimicrobial treatment for cotton samples.

## Figures and Tables

**Table 1:** Flavonoids and phenolic acids contained in *Stellaria media* extract identified by FT–ICR MS.

Compound	Molecular Formula	Calculated Mass (*m*/*z*)		Measured Mass (*m*/*z*)		Mass Accuracy (ppm)	
		ESI+	ESI-	ESI+	ESI-	ESI+	ESI-
Apigenin-7-O-neohesperidoside (Rhoifolin)	C_27_H_30_O_14_	579.170832	-	579.170618	-	−0.369494	-
Apigenin-6,8-di-C-glucoside	C_27_H_30_O_15_	595.165747	593.151194	595.165468	593.149057	−0.468777	−3.602791
Apigenin-6-Arabinosyl-8-galactosyl	C_26_H_28_O_14_	565.155182	563.140629	565.154995	563.140115	−0.330883	−0.912738
Apigenin-6-C-glucoside (Isovitexin)	C_21_H_20_O_10_	433.112923	-	433.112951	-	0.064648	-
Vanillic acid glucoside	C_14_H_18_O_9_Na	353.084303	-	353.083926	-	−1.067734	-
Caffeoylquinic acid	C_16_H_18_O_9_	-	353.087806	-	353.087272	-	−1.512372
Hydroxybenzoic acid	C_7_H_6_O_3_	-	137.024418	-	137.024409	-	−0.065682
Ellagic acid	C_14_H_6_O_8_	303.013544	-	303.014468	-	3.049369	-

**Table 2:** Chromatic parameters of the textile samples (cotton and wool) treated with AgNPs dispersions.

Sample		L*	a*	b*	∆L*	∆a*	∆b*	∆E*
Untreated cotton		93.48	−0.27	3.83	-	-	-	-
Untreated wool		83.96	−0.19	10.17	-	-	-	-
AgNPs	Cotton	87.35	1.40	7.49	−6.12	1.67	3.66	7.33
	Wool	77.91	0.84	8.01	−6.05	1.02	−2.15	6.50

**Table 3:** Bactericidal ratios of the textiles treated with AgNPs dispersions based on counting the CFU of the bacteria strains.

Bacterial Strain	Textile Sample	CFUs/mL on Control Samples	CFUs/mL on Samples Treated with AgNPs Dispersions	Bactericidal Ratio (%)
*Escherichia coli*	Cotton	2.1 × 10^4^	0	100
	Wool	2.8 × 10^4^	0	100
*Saphylococcus aureus*	Cotton	4.5 × 10^4^	4	99.99
	Wool	5.5 × 10^4^	6	99.99
*Bacillus subtilis*	Cotton	2.9 × 10^4^	0	100
	Wool	3.7 × 10^4^	2	99.99

**Table 4:** Images of Petri dishes inoculated with microbial strains and incubated with AgNPs treated textile samples.

Textile Sample	Cotton	Wool
Microbial Strain		
*Escherichia coli*	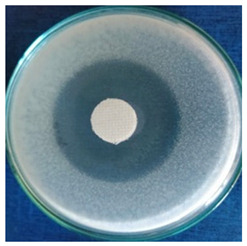	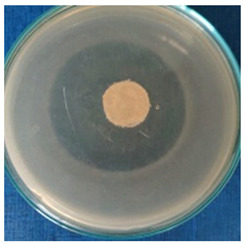
*Saphylococcus aureus*	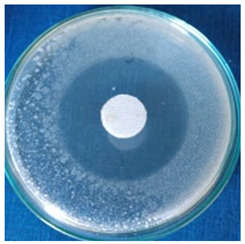	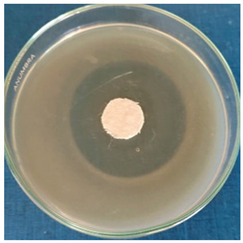
*Bacillus subtilis*	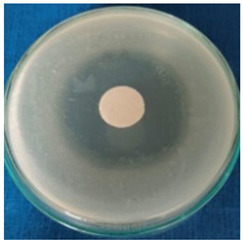	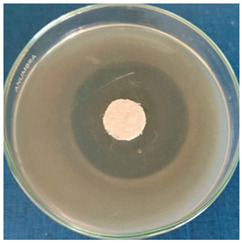
*Penicillium hirsutum*	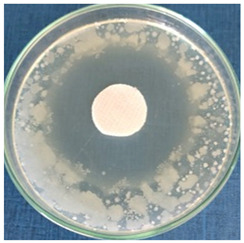	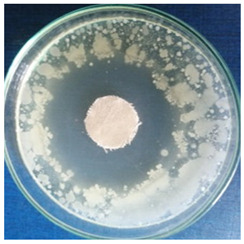

## Data Availability

The data are included in the text.

## Supplementary Materials

The following supporting information can be downloaded at: https://www.mdpi.com/article/10.3390/ma16113924/s1.
