# Supplementary material for: Phytochemical Synthesis of Silver Nanoparticles and Their Antimicrobial Investigation on Cotton and Wool Textiles
Source: Materials (Basel). 2023 May 24;16(11):3924. doi: 10.3390/ma16113924 (PMC10253975; doi:10.3390/ma16113924)
Supplement: Supplementary file 1 [file materials-16-03924-s001.zip › materials-2360696-supplementary.pdf]

S1. Result S1.

1. FT-ICR MS chromatograms

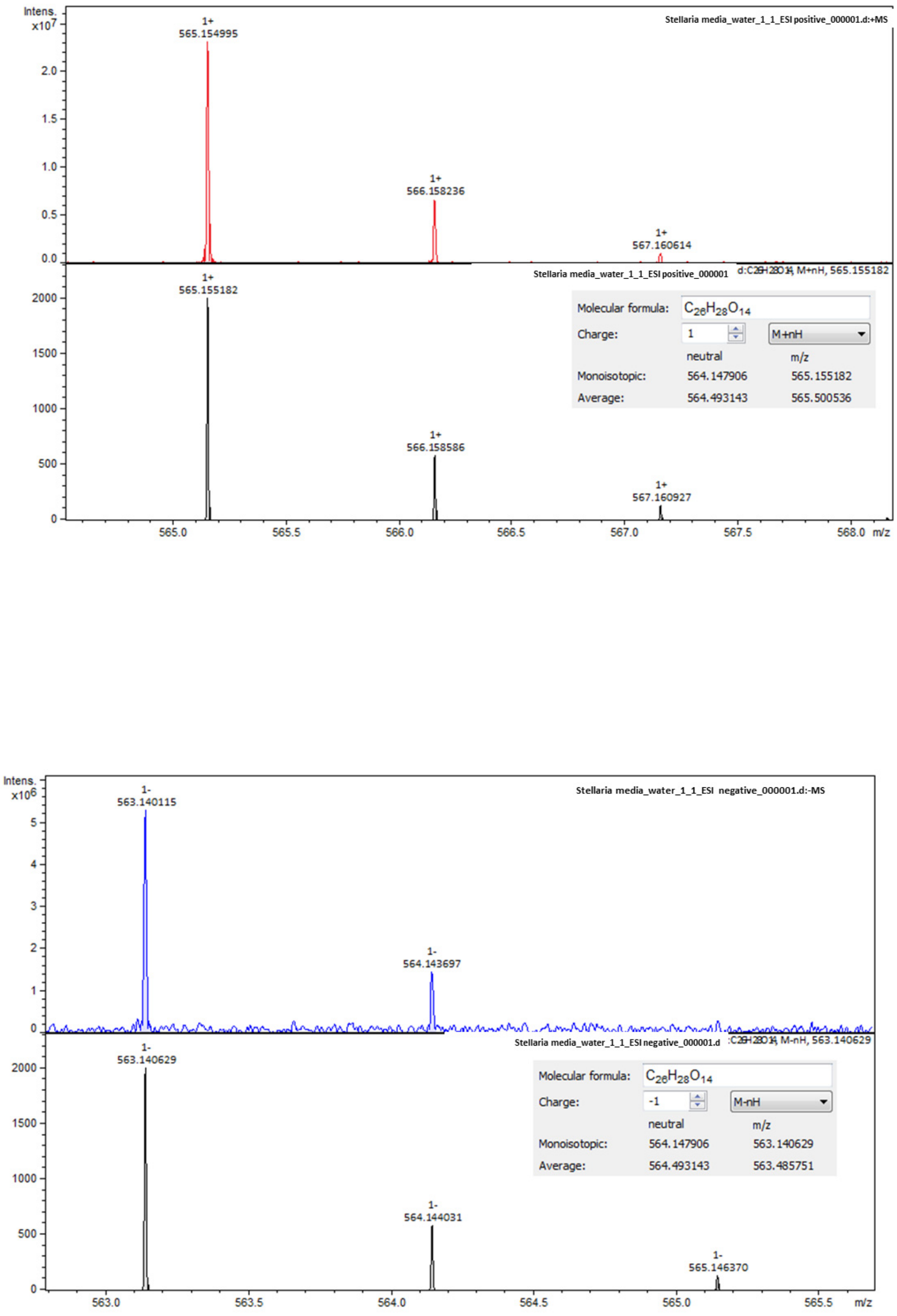

Figure S1. Apigenin-6-Arabinosyl-8-galactosyl ($C_{26}H_{28}O_{14}$) – m/z is 565.15, ESI+ and 563.14, ESI-.

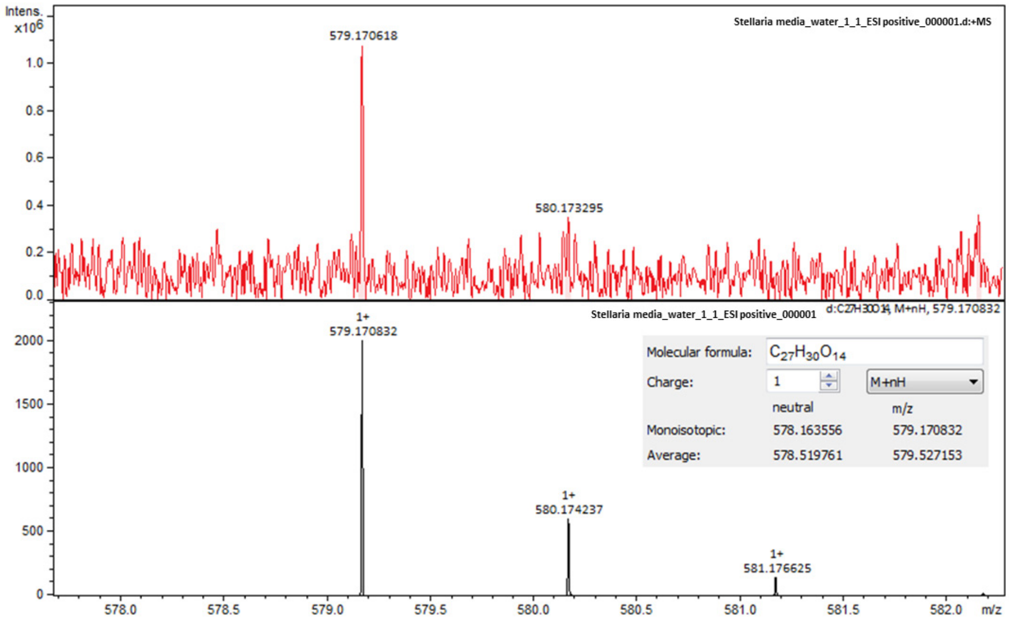

Figure S2. Apigenin-7-O-neohesperidoside (Rhoifolin) ($C_{27}H_{30}O_{14}$) – m/z is 579.170, ESI+

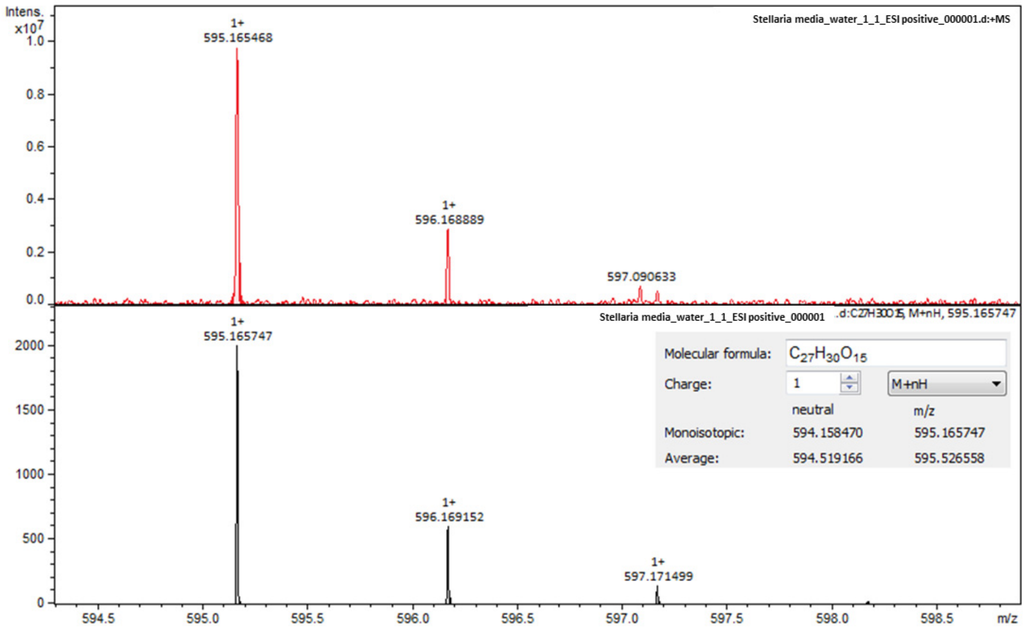

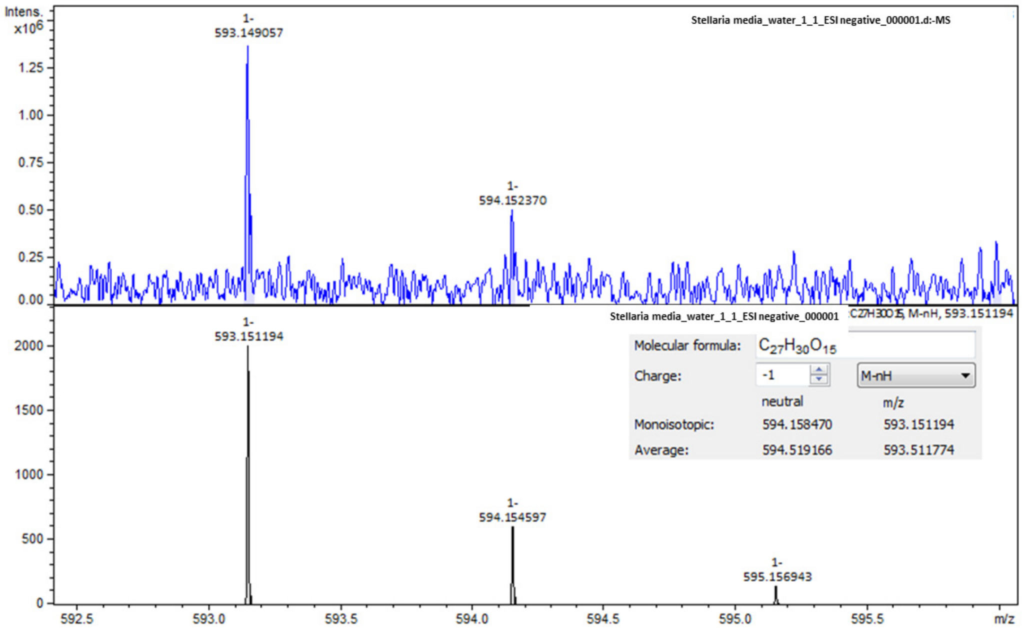

Figure S3. Apigenin 6,8-di-C-glucoside ($C_{27}H_{30}O_{15}$) – m/z is 595.16, ESI+ and 593.15, ESI-.

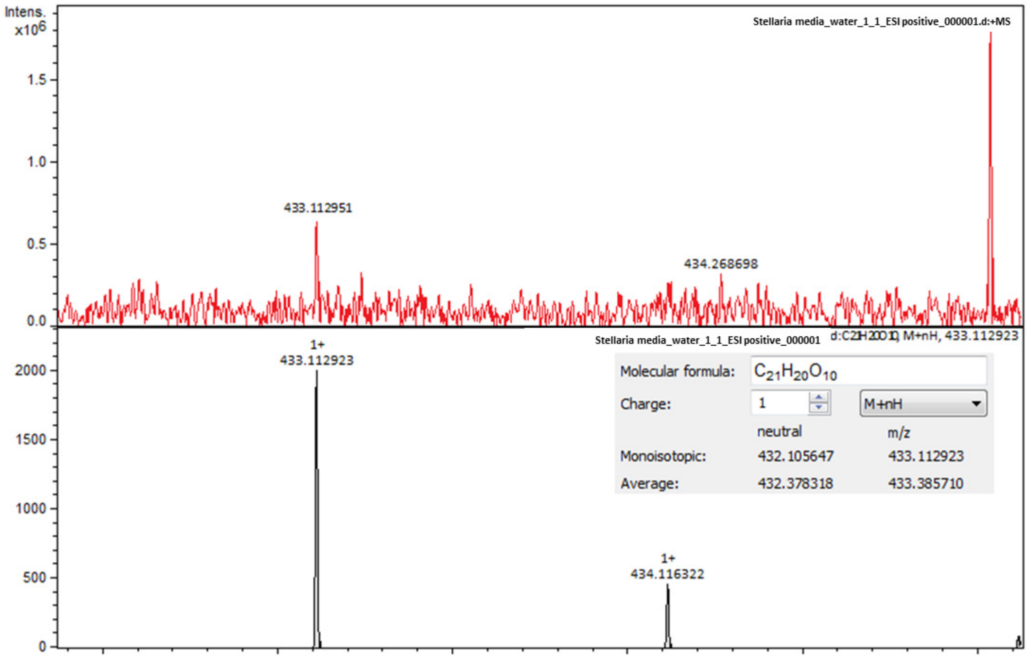

Figure S4. Apigenin-6-C-glucoside (Isovitexin) ($C_{21}H_{20}O_{10}$) – m/z is 433.11, ESI+

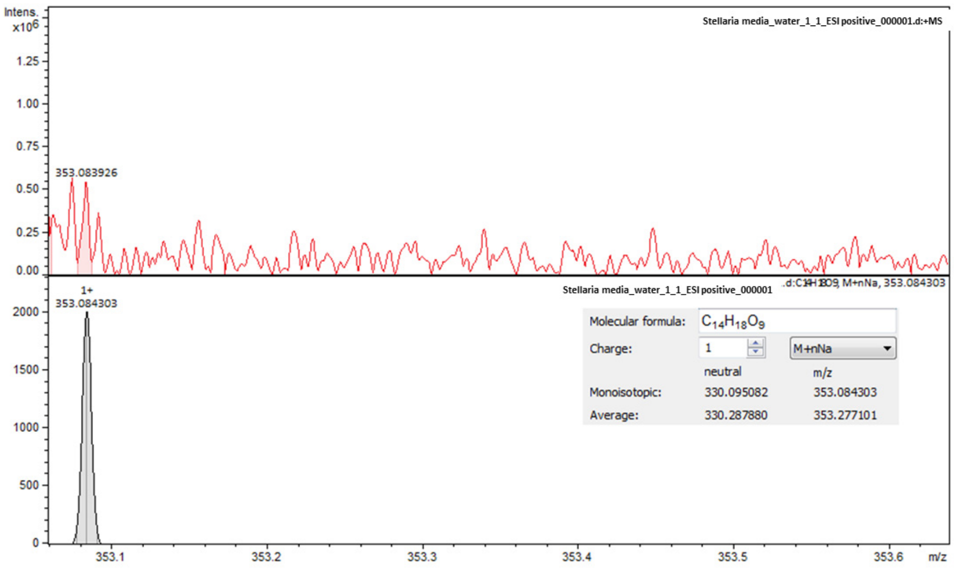

Figure S5. Vanillic acid glucoside ($C_{14}H_{18}O_9Na$) – m/z is 353.08, ESI+

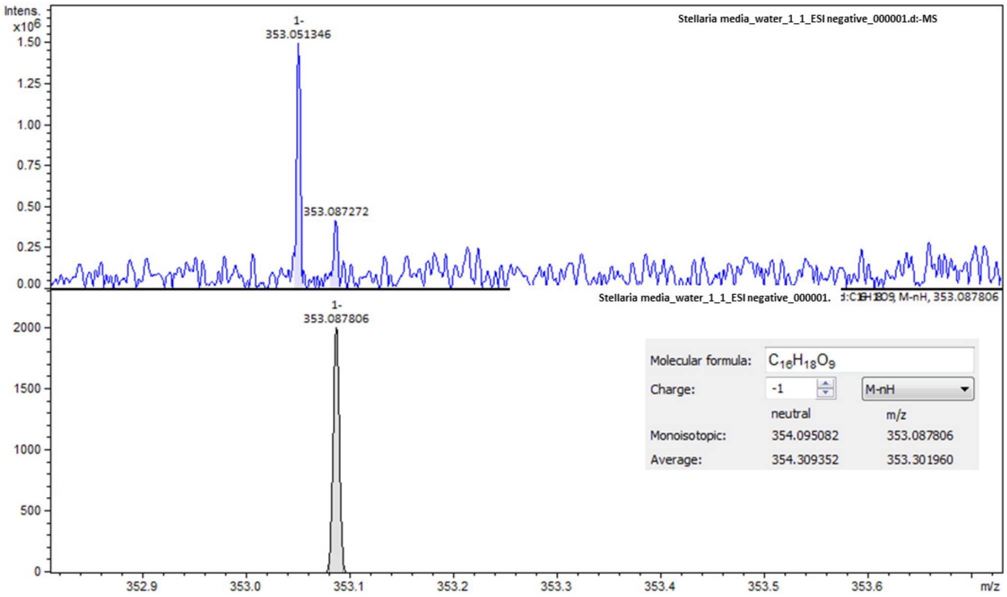

Figure S6. Caffeoylquinic acid ($C_{16}H_{18}O_9$) – m/z is 353.08, ESI-

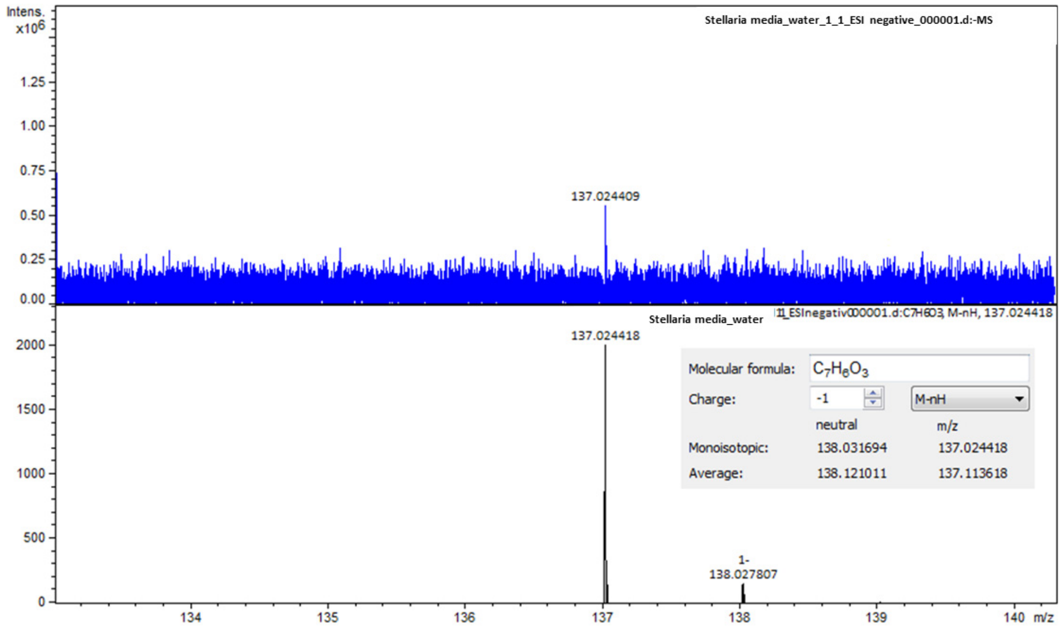

Figure S7. Hydroxybenzoic acid ($C_7H_6O_3$) – m/z is 137.024, ESI-

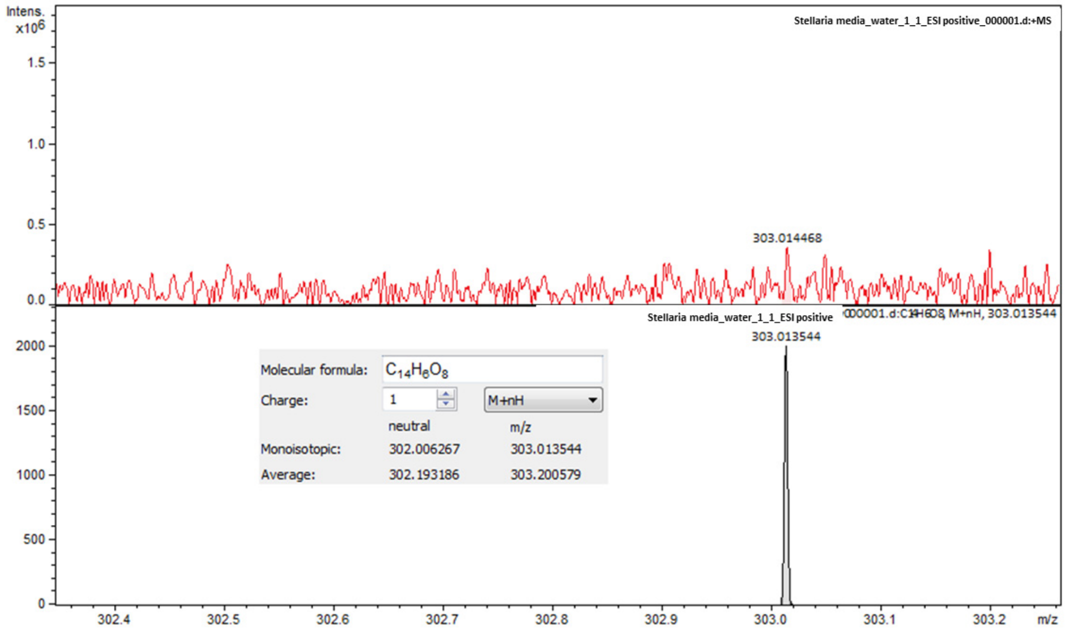

Figure S8. Ellagic acid ($C_{14}H_6O_8$) – m/z is 303.01, ESI+
